# Complete response to the combination of Lenvatinib and Pembrolizumab in an advanced hepatocellular carcinoma patient: a case report

**DOI:** 10.1186/s12885-019-6287-8

**Published:** 2019-11-08

**Authors:** Zhaonan Liu, Xingjie Li, Xuequn He, Yingchun Xu, Xi Wang

**Affiliations:** 1 Department of Oncology, Shanghai Renji Hospital, Shanghai Jiaotong University School of Medicine, Shanghai, 200127 People’s Republic of China; 2 Department of Oncology, the 903rd Hospital of PLA, 14 Lingyin Road, Hangzhou, 310013 China

**Keywords:** Hepatocellular carcinoma, Immunotherapy, Lenvatinib, Pembrolizumab

## Abstract

**Background:**

The majority of patients diagnosed with hepatocellular carcinoma (HCC) have advanced diseases and many are not eligible for curative therapies.

**Case presentation:**

We report a rare case of HCC from a patient who had a complete response (CR) with the use of combination of Lenvatinib and Pembrolizumab. A 63-year-old man presented at the hospital with serious abdominal pain and was found to have a mass with heterogeneous enhancement and with hemorrhage in segment III of the liver after the examination of abdominal computerized tomography (CT) scan. The patient’s history of viral hepatitis B infection, liver cirrhosis and the ɑ-fetoprotein (AFP) level of 14,429.3 ng/ml supported the clinical diagnosis of HCC and laboratory results demonstrated liver function damage status (Child-Pugh class B, Score 8). The patient first received hepatic arterial embolization treatment on 28th November 2017. At this stage supportive care was recommended for poor liver function. In February 2018, combined immunotherapy of Pembrolizumab (2 mg/kg, q3w) and Lenvatinib (8 mg–4 mg, qd) were performed. Nine months following the treatment he had a CR and now, 22 months since the initial treatment, there is no clinical evidence of disease progression. The current overall survival is 22 months.

**Conclusions:**

HCC is a potentially lethal malignant tumor and the combination of immunotherapy plus anti-angiogenic inhibitors shows promising outcome for advanced diseases.

## Background

Hepatocellular carcinoma (HCC) is the fifth leading cause of cancer death in the United States with a 5-year survival rate of 18% for all stages [1] and its incidence rate is rising faster than that of any other cancer in both men and women [2]. Rates of both incidence (18.3 per 100,000) and mortality (17.1 per 100,000) are 2 to 3 times higher in China than those estimated in most other world regions [1]. The major risk factors of HCC vary from region to region. The key determinants in China are chronic hepatitis B virus (HBV) infection and aflatoxin exposure, therefore most cases of HCC in China are at younger ages and with cirrhosis.

Surgery is usually considered the treatment of choice for early disease; however, most patients have locally advanced or metastatic HCC at diagnosis in which case treatments are limited. Furthermore, with the wide range of local regional therapies available to patients with unresectable HCC (uHCC), evidence for favorable systemic therapy for metastatic disease on overall survival (OS) is lacking. Cytotoxic chemotherapies have reported to have low response rates. Oral multi-kinase inhibitors that suppress tumor cell proliferation and angiogenesis in HCC have been approved. Currently, the first line options for uHCC include Sorafenib and Lenvatinib, and second line options are formed by Regorafenib and Cabozantinib. Clinical studies with Nivolumab (Checkmate 040 trial) or Pembrolizumab (KEYNOTE-224) also have promising data for patients with advanced HCC who progressed on or after Sorafenib. The rationale for the combination of Lenvatinib and Pembrolizumab has been illustrated in preclinical studies. Clinical studies of the combination treatment had not been published until June 2018. Preliminary data of the Phase Ib clinical trial of combination treatment (PEM plus LEN) in HCC patients have been published as Keynote-524 in 2019 in the journal of the American Association for Cancer Research (AACR).

To evaluate clinical efficacy of the combination rationale including immune checkpoint inhibitors and multi-kinase inhibitors, we report a case of HCC with poor liver function in the setting of cirrhosis from HBV infection responding dramatically to the combination treatment of Pembrolizumab and Lenvatinib after initial hepatic arterial embolization (HAE) and we hope to explore further study for anti-PD-1 therapy and multi-kinase targeted therapy combination for HCC treatment in the future.

## Case description

**Fig. 1:**
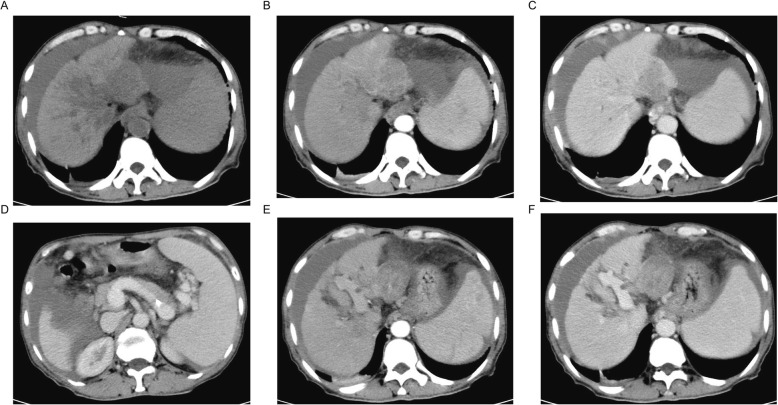
CT of the abdomen showing the segment 3 liver lesions at diagnosis. **a** The characteristics of liver mass in plain scan; **b** Liver mass in arterial phase; **c** Liver mass in portal phase; **d** Multiple collateral circulations; **e** Partial thrombosis of the left portal vein in arterial phase; **f** Partial thrombosis of the left portal vein in portal phase

**Fig. 2:**
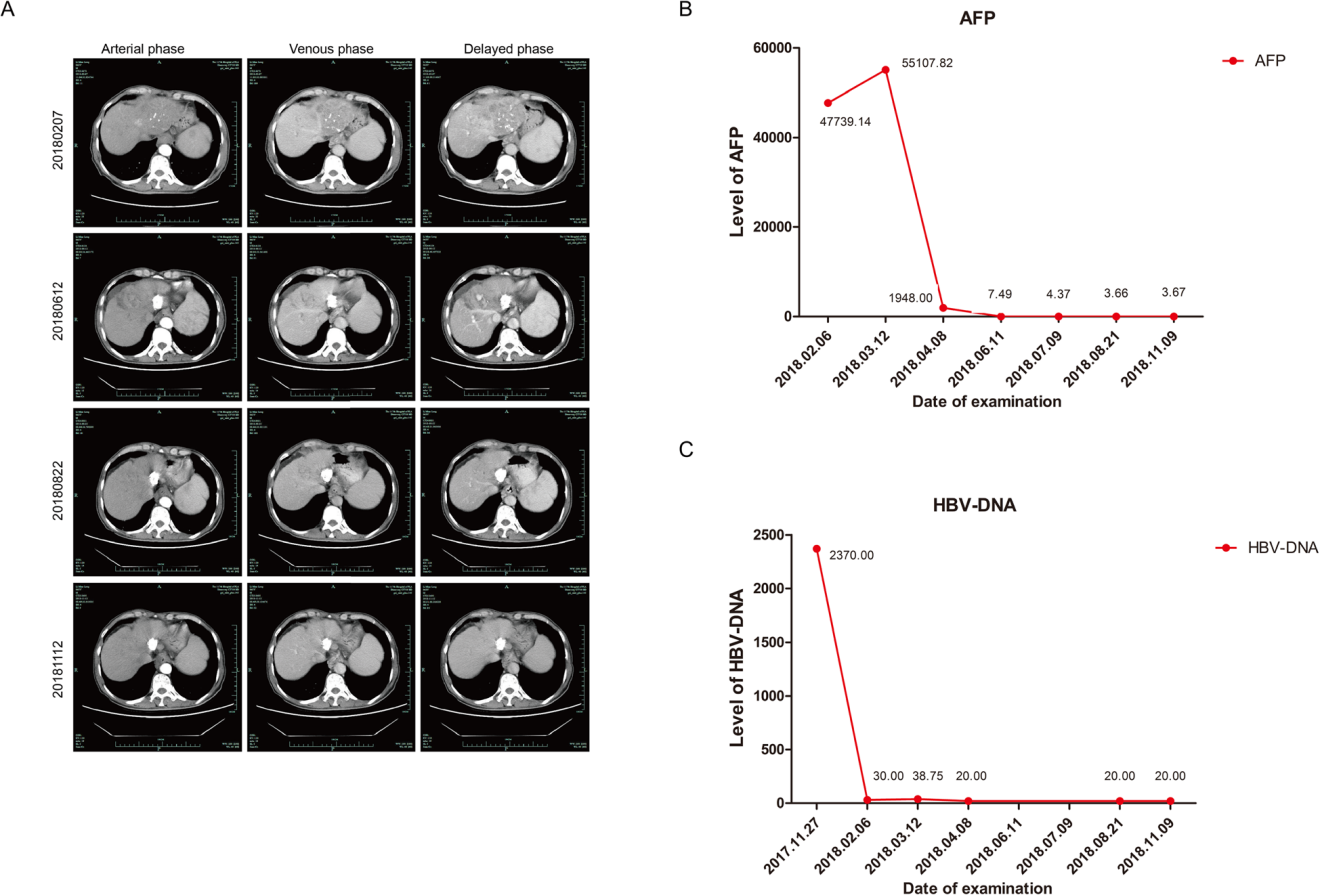
CT of the abdomen showing the segment 3 liver lesions at baseline (Panel A 20180207), 4 months (Panel A 20180612), 6 months (Panel A 20180822) and 9 months (Panel A 20181112) after treatment with Pembrolizumab and Lenvatinib, respectively. Graphical depiction of change in the ɑ-fetoprotein over time (Panel B). Graphical depiction of change in HBV-DNA over time (Panel C)

Our patient, a 63-year-old male with a history of chronic HBV infection for 18 years, presented to the emergency department with severe abdominal pain and flatulence in November 2017. Enhanced abdominal CT showed a heterogeneous irregular mass with the largest measuring up to 5.0 * 3.8 cm in size in segment 3 within the left lobe of liver(Fig. 1a-c). Hepatocellular carcinoma (HCC) with hemorrhage and peritoneal effusion should be considered first. The CT scan also revealed liver cirrhosis, splenomegaly, portal hypertension with multiple collateral circulations (Fig. 1d) and partial thrombosis of the left portal vein (Fig. 1e, f). The laboratory test data revealed serum ɑ-fetoprotein (AFP) was 14,429.3 ng/ml and HBV DNA level of 2.37*10^3 IU/ml. The patient was confirmed with the clinical diagnosis of Barcelona Clinic Liver Cancer (BCLC) C and Child-Pugh class B (Score 8) (Table 1) HCC with the background of cirrhosis secondary to viral B infection. The patient first received HAE and then discharged with an HBV DNA level below 30 IU/ml after antiviral treatment with entecavir. He had radiographic progression 2 months later (Fig. 2a) with poor liver function (Child-Pugh class B 7) (Table 1). For Sorafenib, 400 mg twice daily was only recommended for HCC with liver function of Child-Pugh class A. For lack of an available clinical trial, the patient was prescribed off-label immunotherapy based on the phase I/II data mentioned above (KEYNOTE-224). He was recommended to take Pembrolizumab 100 mg (2 mg/kg, q3w) on Feb. 8th 2018, which was well tolerated. Baseline computed tomography (CT) showed one large liver lesion and extrahepatic hilar lymph node metastasis (Fig. 2a). After one cycle of Pembrolizumab, his AFP increased to 55,107.82 ng/ml compared to 47,739.14 ng/ml before Pembrolizumab was administered (Fig. 2b). The patient was recommended to continue on Pembrolizumab due to the stable clinical status of the patient, and the possibility of pseudoprogression. However, the patient was so worried about the potential progression of his cancer that he started to take Lenvatinib in addition to Pembrolizumab from Mar. 10th for 8 mg per day at home. Adverse effects including grade III diarrhea, grade IV thrombocytopenia according to the common terminology criteria for adverse events (CTCAE4.0) occurred after the administration of Lenvatinib and the liver function was Child-Pugh class C 10 (Table 1). To reduce the adverse effect, Lenvatinib was reduced to 4 mg per day and following this reduction no other treatment-related grade 3 or 4 adverse events were seen. Repeat imaging assessment after 4, 8, 12 cycles of combination treatment showed significant decrease in the size of the liver lesions (2018.06.12), and the subsequent CT scan (2018.08.22) also showed further shrinkage of the tumor and finally a complete response on 12th November 2018, with tumor assessment criteria as mRECIST (Fig. 2 a). AFP was also reduced to a normal range (Fig. 2 b). He remained on treatments with restaging scans every two months which has not shown evidence of recurrence to date. The Progression Free Survival is now 19 months and 22 months have elapsed since the diagnosis of HCC.

**Table 1:** The Child-Pugh class level, HBV DNA level and full blood test analysis corresponding to pembrolizumab and lenvatinib combination therapy

Date	ALB(g/l)	HE	PT	INR	Ascites	T-BIL	Score	HBV-DNA	WBC(10^9/L)	GR(10^9/L)	HGB(g/L)	PLT(10^9/L)
17.11.27	30.4	N	15.3	1.35	Y	16.4	8	2370	1.3	0.58	112	42
18.2.7	31.6	N	15.4	1.3	N	17.3	7	<30	1.45	0.81	118	43
18.3.2	33.0	N	15.6	1.31	N	23.6	7	<30	1.89	0.99	126	48
18.4.8	25	N	20.4	1.73	N	32.7	10	38.7	1.62	0.67	127	21
18.6.11	26	N	16.6	1.4	N	31.1	7	<20	1.21	0.58	110	23
18.7.9	29.7	N	16.5	1.39	N	25.3	7	/	1.26	0.71	113	34
18.7.31	30.3	N	16.6	1.4	N	30.8	7	/	1.42	0.72	120	21
18.8.22	29.2	N	16.2	1.36	N	20.5	7	<20	0.9	0.42	106	25
18.11.9	31.8	N	15.4	1.29	N	21.6	7	<20	1.33	0.69	117	27

*ALB* albumin, *HE* hepatic encephalopathy, *PT* prothrombin time, *INR* international standard ratio, *T-BIL* total bilirubin, *WBC* white blood cell, *GR* granulocyte, *PLT* platelet

*ORR* objective response rate, *AE* adverse event, *DLT* dose limited toxicity, *LEN* Lenvatinib, *PEM* Pembrolizumab, *MTD* maximum tolerance dose, *DOR* duration of response, *dMMR* mismatch repair deficient, *PFS* progression-free survival, *OS* overall survival, *CR* complete response, *PD-L1* programmed cell death ligand 1, *TMB* tumor mutation burden

## Discussion and conclusion

HCC is often diagnosed at advanced stages with limited curative therapy options, leading to a 5-year survival rate of 2% [1]. Conventional systemic therapy with cytotoxic drugs such as doxorubicin and cisplatin achieve low objective response rates (typically < 10%), failing to improve the overall survival (OS) of these patients [3]. FOLFOX4 was compared to doxorubicin in a phase III trial and the PFS was greater for FOLFOX4, but the primary OS endpoint was not met [4].

The launch of sorafenib, a molecular kinase inhibitor, was thought to be a breakthrough in treating uHCC given the results in two randomized-controlled trials (SHARP trial [5] and Asia-Pacific trial [6]) although only 3 months longer OS was found in sorafenib group. Remaining the only FDA-approved therapy for a decade, the benefits of Sorafenib was limited for lack of either therapeutic alternative or second-line treatment for those who are intolerant to Sorafenib [7]. However, during the two-year period from 2017 through 2018, treatment for patients with advanced HCC is dramatically changed by novel multi-target inhibitors approved, Regorafenib, Lenvatinib, Cabozantinib, and single target Ramucirumab or immune checkpoint inhibitors-Nivolumab and Pembrolizumab.

The efficacy of lenvatinib, a multitarget inhibitor, was proved in a phase 3 open-label, multicenter non-inferiority trial, REFLECT study, and the results were published in the Lancet [8]. Median overall survival for lenvatinib was 13.6 months, compared to Sorafenib at 12.3 months (hazard ratio 0.92, 95%CI 0.79–1.06), meeting the study primary criteria for non-inferiority. As a result, the FDA approved Lenvatinib in a first-line setting for patients with unresectable advanced HCC in August 2018 [9].

In recent years, immune checkpoint blockade has brought a paradigm shift in the treatment of a number of malignancies. Various immune checkpoint blocking agents are being tested for their efficacy in HCC. Furthermore, the immune checkpoint blockade of programmed death receptor-1 (PD-1) pathway offers a potential treatment strategy based on the encouraging results of the phase I/II trial of Pembrolizumab (KEYNOTE-224) and Nivolumab (Checkmate 040 trial). KEYNOTE-224 is a non-randomized, multicenter, open-label, phase 2 trial [10], 104 patients with advanced HCC who had progression on or intolerance to Sorafenib received Pembrolizumab 200 mg every 3 weeks. Objective Response rate in 18 patients (17, 95% CI 11–26%) and severe adverse events in 16 of the 104 patients indicate its tolerability and efficacy. Nivolumab, another anti-PD-1 antibody, was assessed in the Checkmate 040 trial for patients with advanced HCC. The objective response rate was about 20%, the disease control rate was 64% and the median duration of response is 17 months for Sorafenib-naïve patients and 19 months for patients who had been previously treated with Sorafenib [11]. The FDA approved the use of Nivolumab in 2017 for patients with HCC who progressed on or after Sorafenib and the liver function is Child-Pugh A or B^9^. A phase III RCT, Checkmate 459, in which nivolumab is being compared to Sorafenib as first-line treatment in patients with advanced HCC is currently in progress (NCT02576509).

Currently, the first line options for uHCC include sorafenib and lenvatinib, and second line options are formed by Regorafenib, Nivolumab, Pembrolizumab, and Cabozantinib [9]. The combination of Lenvatinib and Pembrolizumab is a novel but potent competitor for the future gold standard in the systemic treatment of uHCC. Lenvatinib was proved to be an immunomodulator in tumor microenvironment [12] while PD-1 antibody blocks the co-inhibitory signals and unlocks the negative regulation of the immune response [13]. In the hepa1–6 hepatocellular carcinoma model, treatment with lenvatinib decreased the proportion of monocytes and macrophages population and increased that of CD8+ T cell populations, indicating the immunomodulatory activity of Lenvatinib [14]. This combination inhibited cancer immunosuppressive environments induced by tumor-associated macrophages and Tregs, reducing the levels of TGF-β and IL-10, the expression of PD-1, and the inhibition of Tim-3, and thereby triggering anticancer immunity mediated by immunostimulatory cytokines such as IL-12 [15]. Therefore, further investigations for the combination treatment of Lenvatinib and Pembrolizumab are warranted to provide its efficacy data in clinical trials. We conducted a thorough English literature search on PubMed using the search terms ‘anti-PD-1,’ ‘pembrolizumab’ or ‘nivolumab,’ ‘lenvatinib’ and ‘hepatocellular cancer’, ‘HCC,’ or ‘hepatoma’. There are no published data from randomized controlled trials in HCC; however, an ongoing open-label phase 1b trial (KEYNOTE 524, NCT03006926) (Table 2), is the only study on the combination treatment of Lenvatinib plus Pembrolizumab registered on clinicaltrial.gov currently. Preliminary results were presented in the form of a poster at the American Society of Clinical Oncology Annual Meeting in 2018. The study was designed into 2 parts, including dose limiting toxicity (DLT) evaluation and expansion parts to demonstrate its safety and efficacy, respectively. Patients of uHCC with BCLC stage B or C, Child-Pugh Class A and no other systemic treatment (including Sorafenib) were enrolled for tolerability and efficacy (through CR or PR) assessments. They received Lenvatinib 12 mg (body weight over 60 kg) or Lenvatinib 8 mg (body weight less than 60 kg) orally once-daily and Pembrolizumab 200 mg IV once 3 weeks as the standard regimen. No dose limited toxicities were reported in Part 1 of the study and 3 of the 24 deaths were considered treatment-related in Part 2. The most common treatment-emergent adverse events for any grades were decreased appetite (53.3%), hypertension (53.3%), diarrhea (43.3%) and fatigue (40.0%). Objective response rate, assessed by mRECIST is 11 out of 26 (42.3, 95%CI 23.4–63.1), including 4 cases with unconfirmed responses. The estimated median duration of progression-free survival was 9.69 months. Data above demonstrate the tolerability and encourage the antitumor activity of the combination therapy. Combination therapy of Lenvatinib and Pembrolizumab is a novel and potent therapeutic regimen for the uHCC. Although the clinical trial of this combination is still in phase 1b and ongoing, preliminary results are encouraging for its safety and efficacy. The eligibility criteria for this trial includes BCLC stage B (not applicable for transcatheter arterial chemoembolization (TACE)) or C, Child-Pugh class A, ECOG performance status 0–1, which means the preserved liver function is good among the patients enrolled. There is no report on the efficacy of this combination for patients whose Child-Pugh at class B with cirrhosis at the decompensation stage. Our case was diagnosed of HCC with ascites, cirrhosis, splenomegaly, and portal vein hypertension at his first visit at the emergency department, indicating the deterioration of the liver function. Irregular Lenvatinib 8 mg–4 mg (lower dose because of intolerance of the adverse effect) usage and 7 cycles of Pembrolizumab 100 mg (2 mg/kg) injection with an interval of 3 to 4 weeks dramatically decreased the AFP from 47,739.14 ng/ml to the normal range and reached CR according to mRECIST. The PFS is 19 months and 22 months had elapsed since the diagnosis of HCC. So, the responses appear to be durable. Further follow-up for this patient is ongoing. The complete response of Lenvatinib in REFLECT trial or Pembrolizumab in KEYNOTE-224 trial is both 1%, so even for patients with good ECOG status and enough liver function, CR is not very common. The great success in this case demonstrates the possible feasibility of the combination treatment in uHCC at decompensate stage ((BCLC C and Child-Pugh class B Score 8) for patients who are not suitable for sorafenib due to poor liver function. Standard combination and sequencing of the therapy need to be established with deeper insight into the rationale of combined action and further RCTs. What’s more, the patients enrolled in, for most of the cases, present with preserved liver function, while the advanced HCC patients in real clinical phase may have a much worse performance. Whether they can tolerate the combination treatment is still unknown and the clinical trials won’t take the risk to enroll these patients. No life-threatening adverse events were found in our patient according to treatment due to a decrease in the dosage of both PEM and LEN. Notably, there are many ongoing trials to evaluate the safety and efficacy of checkpoint inhibitors and Lenvatinib in solid tumors (Table 2), and a subgroup of NCT02501096 (Table 2) showed anti-tumor activity in patients with advanced recurrent endometrial cancer with a safety profile that was similar to those previously reported for Lenvatinib and Pembrolizumab monotherapies, apart from an increased frequency of hypothyroidism [16]. Serum level of HBV DNA should be considered for surveillance of HBV-infected patient who receive immunotherapy. The patient had a high HBV DNA level of 2.37*10^3 IU/ml at first diagnosis followed by HBV DNA level below 30 IU/ml after antiviral treatment with entecavir and below 20 IU/ml during follow up.

**Table 2:** Ongoing clinical trials with immune checkpoint blockade pembrolizumab and lenvatinib in solid tumors

NCT number	Number of patients	Cancer type	Trial phase	Line of therapy	Gene or protein detection	Primary endpoints	Current status
NCT03609359	29	Advanced Gastric Cancer	2		/	ORR	recruiting
NCT03006887	6	Transitional Cell Carcinoma Renal Cell Carcinoma Clear Cell Renal Cell Carcinoma	1b		/	1.AE 2.DLT	active, not recruiting
NCT02501096	329	Tumors involving non-small cell lung cancer, renal cell carcinoma, endometrial cancer, urothelial cancer, squamous cell carcinoma of the head and neck, or melanoma	1b(LEN)/2(PEM)	Salvage therapy	/	1.MTD (phase 1b) 2.ORR 3.DLT	recruiting
NCT03006926	104	Hepatocellular Carcinoma	1b		/	1.AE 2.DLT 3.ORR 4.DOR by Mrecist and RECIST 1.1 based on IIR analysis	recruiting
NCT03797326	180	Advanced Solid Tumors Triple Negative Breast Cancer Ovarian Cancer Gastric Cancer Colorectal Cancer Glioblastoma Biliary Tract Cancers	2	Salvage therapy	dMMR for Colorectal Cancer	1.ORR 2.Percentage of AE 3.Percentage of Discontinue Study Treatment	not yet recruiting
NCT03776136	100	Advanced Melanoma	2		/	ORR	recruiting
NCT03820986	660	Malignant Melanoma	2(PEM)/3(LEN)	first-line	/	1.PFS 2.OS	not yet recruiting
NCT02973997	60	Thyroid Gland Carcinoma	2	metastasis	/	1.CR rate 2.Confirmed response rate	recruiting
NCT03829332	620	Non-small Cell Lung Cancer	3	first-line	PD-L1 ≥ 1%	1.PFS 2.OS	not yet recruiting
NCT03713593	750	Hepatocellular Carcinoma	3	first-line	/	1.PFS 2.OS	recruiting
NCT03517449	780	Endometrial Neoplasms	3		MMR	1.PFS 2.OS	recruiting
NCT03321630	24	GastroEsophageal Cancer	2	salvage therapy	correlative biomarker studies.	1.ORR 2.OS	recruiting
NCT03829319	726	Nonsquamous Non-small Cell Lung Cancer	3	first-line	PD-L1	Part 1: DLT AE Part 2: PFS OS	not yet recruiting
NCT02811861	1050	Renal Cell Carcinoma	3	first-line	/	PFS	recruiting
NCT03516981	192	Advanced Non-Small Cell Lung Cancer	2		Gene expression profile and TMB	ORR	recruiting

There are a few potential factors discussed to estimate prognosis, including emergent adverse AFP, PD-L1, requiring further evidence to verify their potency in this novel combination treatment. AFP (over 400 ng/mg) and PD-L1 (over 1%) are reviewed as potential biomarkers to estimate the prognosis in certain treatment regimen of HCC patients whereas neoantigen, tumor mutational burden, and interferon gamma need further investigation [17]. Notably, our patient lacked diagnosis that was confirmed by pathology and without data of PD-L1 expression, we recommended the gene examination of peripheral blood ctDNA, but the patient refused to pay for this additional testing. Even without the benefit of PD-L1 expression data he still got a good response for this combination treatment, which raises questions about the value of PD-L1, dMMR, or TMB testing as a biomarker in HCC when immunotherapy is combined with other therapies. Whether this great success could be duplicated is still unknown. Exploration of the possible indicators for the combination and prognosis estimating factors are the foundation for a wider application.

Other anti-angiogenic/immunotherapy combinations are also currently popular subjects for research. An Atezolizumab (atezo) and Bevacizumab (bev) regimen was well-tolerated and had a manageable safety profile in patients with microsatellite-high (MSI-high) metastatic colorectal cancer (mCRC), according to results of a preliminary clinical evaluation presented at the 2017 Gastrointestinal Cancers Symposium. The overall response rate was 40% using the RECIST criteria and 30% via immune-related response criteria (irRC) [18]. Another study of Regorafenib plus nivolumab in patients with advanced gastric or colorectal cancer (REGONIVO, EPOC1603) was published at ASCO 2019, the ORR and DCR was 40 and 88% respectively [19]. Studies of Bevacizumab in combination with Atezolizumab in patients with untreated melanoma brain metastases (NCT03175432), NSCLC (NCT03836066), recurrent or metastatic squamous-cell carcinoma of the head and neck (NCT03818061) are ongoing.

In summary, what we could learn from this case is that the combination treatment of LEN and PEM with decreased dose and prolonged interval may be tolerable and effective among unresectable HCC patients with cirrhosis (those with hepatitis B infection) at a decompensated stage. While our case highlights some important aspects of the use of combination therapy, especially in HCC cases that lacked definitive expression of PD-L1 or dMMR, the potential side effects of the combination treatment should be highly concerned, and fully discussed with the patients in clinical practice. Lenvatinib plus Pembrolizumab may present a new potential treatment option for the sub-population. However, its efficacy and safety need further investigation in a randomized phase 3 study.

## Data Availability

All relevant data and diagnostic results are contained. The raw data is not made available in consideration of confidentiality.

## Abbreviations

AE: adverse event
AFP: ɑ-fetoprotein
ALB: Albumin
BCLC: Barcelona Clinic Liver Cancer
CR: Complete response
CT: Computerized tomography
ctDNA: circulating tumor DNA
DLT: Dose limited toxicity
dMMR: mismatch repair deficient
DOR: Duration of response
ECOG: Eastern Cooperative Oncology Group
GR: Granulocyte
HAE: Hepatic arterial embolization
HBV: Hepatitis B virus
HCC: Hepatocellular carcinoma
HE: Hepatic encephalopathy
IL-10: interleukin-10
INR: international standard ratio
IV: intravenous
LEN: Lenvatinib
mRECIST: modified response evaluation criteria in solid tumors
MTD: Maximum tolerance dose
ORR: Objective response rate
OS: Overall survival
PD-1: Programmed death receptor-1
PD-L1: Programmed cell death ligand 1
PEM: Pembrolizumab
PFS: progression-free survival
PLT: Platelet
PT: Prothrombin time
TACE: Transcatheter arterial chemoembolization
T-BIL: Total bilirubin
TGF-β: Transforming growth factor-β
TIM-3: T cell immunoglobulin domain and mucin domain-3
TMB: Tumor mutation burden
uHCC: unresectable hepatocellular carcinoma
WBC: White blood cell
